# *Zehneria grandibracteata* (Cucurbitaceae), an overlooked new species from western Kenyan forests

**DOI:** 10.3897/phytokeys.165.57399

**Published:** 2020-10-28

**Authors:** Neng Wei, Zhi-Xiang Zhong, David Kimutai Melly, Solomon Kipkoech, Benjamin Muema Watuma, Veronicah Mutele Ngumbau, Peris Kamau, Guang-Wan Hu, Qing-Feng Wang

**Affiliations:** 1 CAS Key Laboratory of Plant Germplasm Enhancement and Specialty Agriculture, Wuhan Botanical Garden, Chinese Academy of Sciences, Wuhan, CN-430074, China; 2 Sino-Africa Joint Research Center, Chinese Academy of Sciences, Wuhan, CN-430074, China; 3 University of Chinese Academy of Sciences, Beijing, CN-100049, China; 4 East Africa Herbarium, National Museums of Kenya, P.O. Box 451660-0100, Nairobi, Kenya

**Keywords:** East Africa, Flora of Kenya, phylogeny, taxonomy, *Zehneria scabra*

## Abstract

*Zehneria grandibracteata*, a new species of Cucurbitaceae from western Kenya, is described here, based on morphological and molecular data. It has long been misidentified as the widely-distributed species *Z. scabra*. However, it differs by its ovate leafy probract at the base of the inflorescences, subglabrous condition of the entire plant, shorter receptacle-tube and filaments, as well as denser and sessile inflorescences. Furthermore, the molecular phylogenetic analysis of *Zehneria*, based on nrITS sequences, further supports the argument that *Z. grandibracteata* should be segregated from *Z. scabra*.

## Introduction

*Zehneria*Endlicher (1833: 69) is a genus of Cucurbitaceae. It contains over 60 species, which are mainly distributed in tropical and subtropical Africa, Madagascar and south-eastern Asia (Schaefer and Renner 2011a; Dwivedi et al. 2018). *Zehneria* is characterised by male flowers largely with the three stamens all 2-thecate, the thecae ± erect, straight or little curved (Simmons and De Wilde 2000; Schaefer and Renner 2011a). De Wilde and Duyfjes (2006a, b, 2009a, b) split several genera from *Zehneria* s.l. (in the sense of Jeffrey), with only the type species, *Zehneria baueriana*Endlicher (1833: 69) remaining in *Zehneria* s.s.. Besides, De Wilde and Duyfjes (2006a) proposed morphological characters including leaf drying colour, stamen insertion, presence or absence of staminode, presence or absence of probract and shape of stigmatic lobes, disc and seed, in their circumscription of *Zehneria* s.s. and the related genera. Nevertheless, this treatment is not supported by the molecular phylogeny inferred by Schaefer et al. (2009), Schaefer and Renner (2011a, b) and Dwivedi et al. (2018), who argued against over-splitting of the group. East Africa has been recognised as a neglected diversity centre for *Zehneria* (Wei et al. 2017), with several new taxa discovered and named in recent years (Zhou et al. 2016; Wei et al. 2017; Watuma et al. 2019; Ngumbau et al. 2020). Besides, Africa was also referred to as the origin centre (Schaefer et al. 2009; Dwivedi et al. 2018), followed by recent long-distance dispersal to other continents and islands.

During field investigations of the Kenyan flora in 2016, a *Zehneria* species with evident leafy probracts attracted the authors’ attention for the first time. Herbarium specimens had been identified as *Z. scabra* Sond. in Harvey and Sonder (1862: 486), a widespread species with great morphological variability. In the following years, more specimens were collected and detailed morphological studies were conducted. Measurements of morphological characters, as well as molecular phylogenetic analysis, based on nrITS, all support the segregation of this *Zehneria* from *Z. scabra*. Hence, we describe it as *Z. grandibracteata* below.

## Materials and methods

### Morphology

Specimens of East African *Zehneria* deposited in the herbaria of K, EA and HIB were studied, as well as relevant digitised specimens from online databases, including specimens from the herbaria B, BR, BM, E and P (herbarium acronyms follow Thiers (2020)). Morphological measurements of the details given in the description are based on living materials during the field trips, except tendrils and seeds confirmed by specimen observations at herbaria. The detailed morphological comparison between *Z. scabra* and our collection was initially made. Given *Z. longiflora* G.W. Hu & Q.F. Wang in Wei et al. (2017: 89) has largely overlapped the distribution area with our collection, as well as the great similarity with the latter, *Z. longiflora* was also included for morphological comparison.

### Molecular phylogeny

Aiming to delimitate the phylogenetic position of our *Zehneria* collections, a total of 63 sequences were used to infer a phylogenetic tree. Amongst these sequences, 60 accessions representing 38 *Zehneria* species were included and another three accessions from *Cucumis*, *Coccinia*, *Benincasa* were treated as outgroups, according to Schaefer et al. (2009) and Dwivedi et al. (2018). Nineteen sequences of African *Zehneria* species were newly generated in this study, while the other sequences were downloaded from GenBank. The source of the materials and the corresponding GenBank accession numbers were given in Table 1. Total genomic DNA was extracted from silica gel-dried material using a modified CTAB protocol (Doyle and Doyle 1987) (see Suppl. material 1). The primers of nrITS region were obtained from White et al. (1990). PCR amplification, sequencing and data analysis were performed according to Dwivedi et al. (2018). Forward and reverse sequences were manually checked and edited where necessary. Sequences were aligned by MAFFT v. 7 (Katoh and Standley 2013). Gblocks (Talavera and Castresana 2007) was used to trim with the default setting to remove any ambiguous alignment. Additionally, these alignments were visually inspected in Geneious 8.0.2 (Kearse et al. 2012) and manually adjusted where needed. The best-fit model for Bayesian Inference (BI) and Maximum Likelihood (ML) analyses was estimated by ModelFinder (Kalyaanamoorthy et al. 2017) under the Bayesian Information Criterion (BIC). ML analyses were inferred by IQ-TREE v.1.6.8 (Nguyen et al. 2015) under the Ultrafast bootstrapping algorithm (Guindon et al. 2010) with 1000 bootstrap replicates. BI analyses were performed with MrBayes 3.2.7 (Ronquist et al. 2012). Two independent Markov Chain Monte Carlo analyses (MCMC) were run with four simultaneous chains of 10 million generations sampling one tree every 1000 generations with the initial 25% discarded as burn-in. The remaining trees were then used to construct majority-rule consensus trees. The average deviation of split frequencies was verified by reaching a value below 0.01 at the end of MCMC analyses. The effective sample sizes (ESS) for all parameters and statistics were assessed using Tracer version 1.7.1 (Rambaut et al. 2018). The phylogenetic tree was visualised using the online tool iTOL (Letunic and Bork 2007).

**Table 1.** GenBank accession numbers for sequence data used in this study.

Species and specimen-voucher	Accession No.
*Benincasa hispida*, *Renner et al. 2760* (M)	KJ467162
*Coccinia grandis*, *DeWilde & Duyfjes 22270* (L)	HQ608207
*Cucumis melo*, *Mitchell & Schaefer 68* (TUM)	KY434575
*Neoachmandra boholensis*, *Ramos 2-107/37215* (US)	KY523290
*Neoachmandra capillacea*, *Achigan-Dako 07nia757*	AM981144
*Neoachmandra capillacea*, *Wieringa 11246* (M)	KY523291
*Neoachmandra cunninghamii*, *Telford 12489* (M)	KY523292
*Neoachmandra filipes*, *Brass 31994* (US)	KY523293
*Neoachmandra gilletii*, *De Wilde 11246* (L)	KY523280
*Neoachmandra hallii*, *Achigan-Dako 91sn003*	AM981143
*Neoachmandra hermaphrodita*, *Phonsena 440938* (K)	KY523281
*Neoachmandra japonica*, *Su EM0045T001*	MK771856
*Neoachmandra japonica*, *Zhang 1518* (M)	KY523294
*Neoachmandra leucocarpa*, *Junghuhn s.n.* (U)	KY523295
*Neoachmandra odorata*, *He s.n.* (K)	KY523307
*Neoachmandra odorata*, *Wallich 6706* (M)	KY523297
*Neoachmandra pentaphylla*, *Guillaumin 8611* (US)	KY523286
*Neoachmandra pentaphylla*, *McKee 3504* (US)	KY523300
*Neoachmandra samoensis*, *Sykes 170278* (L)	KY523301
*Neoachmandra samoensis*, *Whistler W2908* (B)	MG680626
*Neoachmandra thwaitesii*, *Pallithanam 3637* (BLAT)	KY523314
*Neoachmandra wallichii*, *Fujikawa 053262* (TUM)	KY523310
*Zehneria anomala*, *Gilbert 1681* (EA)	MT733849
*Zehneria anomala*, *Gillett 16503* (M)	KY523289
*Zehneria baueriana*, *McKee 38396* (GH)	KY523288
*Zehneria baueriana*, *Sykes 533* (US)	KY523284
*Zehneria bodinieri*, *Dwivedi 1004* (DUH)	KY523266
*Zehneria bodinieri*, *Tanaka 080913* (MBK)	KY523267
*Zehneria emirnensis*, *Mitchell & Schaefer 25* (TUM)	KY523268
*Zehneria grandibracteata*, *SAJIT 6670* (EA/HIB)	MT733851
*Zehneria grandibracteata*, *SAJIT 6966* (EA/HIB)	MT733852
*Zehneria grandibracteata*, *SAJIT 6968* (EA/HIB)	MT733850
*Zehneria guamensis*, *Perlman 14* (US)	KY523273
*Zehneria longiflora*, *SAJIT 6669* (EA/HIB)	MT733853
*Zehneria longiflora*, *SAJIT 6672* (EA/HIB)	MT733854
*Zehneria marlothii*, *Merxmueller & Giess 30031* (M)	KY523283
*Zehneria maysorensis*, *CALI 10625*	KY523386
*Zehneria maysorensis*, *Dwivedi 1002* (DUH)	KY523256
*Zehneria microsperma*, *Loveridge 64* (GH)	KY523274
*Zehneria minutiflora*, *SAJIT 8861* (EA/HIB)	MT733855
*Zehneria minutiflora*, *Stolz 1139* (M)	KY523296
*Zehneria monocarpa*, *SAJIT 7172* (EA/HIB)	MT733856
*Zehneria monocarpa*, *SAJIT 7173* (EA/HIB)	MT733857
*Zehneria oligosperma*, *Luke 11710* (EA)	MT733858
*Zehneria pallidinervia*, *Holstein 52* (M)	KY523287
*Zehneria pallidinervia*, *SAJIT 6241* (EA/HIB)	MT733859
*Zehneria perpusilla*, *Santapau 13074* (BLAT)	KY523255
*Zehneria perrieri*, *Mitchell & Schaefer 10* (TUM)	KY523270
*Zehneria pisifera*, *Hoogland & Pullen 5926* (GH)	KY523275
*Zehneria polycarpa*, *Mitchell & Schaefer 36* (TUM)	KY523276
*Zehneria racemosa*, *Mendes 1841* (M)	KY523298
*Zehneria scabra*, *Schaefer 05/317*	HQ202009
*Zehneria scabra*, *SAJIT 6501* (EA/HIB)	MT733860
*Zehneria scabra*, *SAJIT 6554* (EA/HIB)	MT733861
*Zehneria scabra*, *SAJIT 6736* (EA/HIB)	MT733863
*Zehneria scabra*, *SAJIT 6873* (EA/HIB)	MT733865
*Zehneria scabra*, *Schaefer s.n.*	KY523278
*Zehneria scrobiculata*, *Bolus 11558* (M)	KY523285
*Zehneria scrobiculata*, *Schimper 164* (M)	KY523299
*Zehneria tahitensis*, *Sachet 2662* (US)	KY523313
*Zehneria tridactyla*, *Espirito 3053* (M)	KY523321
*Zehneria tuberifera*, *SAJIT-6350* (EA/HIB)	MT733866
*Zehneria tuberifera*, *SAJIT-W0044* (EA/HIB)	MT733867

## Results

### Morphological comparison

The Table 2 distinguishes morphological characters of these three species, mainly based on Jeffrey (1967, 1978), Wei et al. (2017) and observations on specimens. Our collection can be readily recognisable by its large leafy probract. Besides, it also differs from the other two species by morphological characters including thick stem, subglabrous leaf blade, sessile inflorescence and size of perianth, pedicel, filament, style and fruit.

**Table 2.** Dissimilar characters to distinguish *Zehneria grandibracteata*, *Z. longiflora* and *Z. scabra*, based on Jeffrey (1967, 1978), Wei et al. (2017) and own observations.

Character	*Z. grandibracteata*	*Z. scabra*	*Z. longiflora*
Stem	Thick, up to 2.5 cm in diam., subglabrous	Thick, up to 1.5 cm in diam., puberulous	Thin, up to 0.8 cm in diam., subglabrous
Leaf blade	Membraneous, deeply cordate to subtruncate at the base, subglabrous, with sparsely scabrid setulose on both sides	Membraneous to subcoriaceous, deeply cordate to subtruncate at the base, puberulous on both sides or sparsely scabrid-setulose on the veins beneath	Slightly fleshy, membraneous, subglabrous, cordate to subtruncate at the base, with sparsely scattered bristles on adaxial surface only
Male inflorescence	Sessile, subumbelliform	Subumbelliform or shortly racemiform sessile or pedunculate axillary clusters	Sessile or pedunculated, subumbelliform or racemiform
Probract	Well-developed, leafy, ovate, up to 18 × 12 mm, incurved, beak-like, persistent	Linear, hooked or curly, minute, caduceus	Linear, hooked or curly, less than 10 mm long, minute, caduceus
Perianth	Receptacle-tube 1.8–3 mm long, hairy only on inner surface, petal lobes ca. 1.8 mm long	Receptacle-tube 2.0–5.5 mm long, hairy on both inner and outside surface, petal lobes 1.5–3.5 mm long	Receptacle-tube 6.0–7.5 mm long, hairy only on inner surface, petal lobes 2.0–3.0 mm long reflexed
Pedicle	3–12 mm long in male, 4–6 mm long in female	1.5–10 mm long in male, 0.4–11.0 (20.0) mm long in female	4–20 mm long in male, 8–25 mm long in female
Filament length	ca. 1.5 mm	1–2.5 mm	ca. 3.5 mm
Style length	2–3.5 mm long, stigma ca. 1.5 mm in diam.	2–4 mm long, stigma ca. 2 mm in diam.	6–7 mm long, stigma ca. 2 mm in diam.
Ovary	Glabrous, subglobose, with neck up to 1 mm long	Puberulous, subglobose to fusiform to beaked, with neck up to 2 mm long	Glabrous, subglobose, with neck up to 3.5 mm long
Fruit	2–16 in clusters, sparsely covered with tiny protuberances, subglobose, 8–10 mm in diam.	1–10 in clusters, usually glabrous, globose, 8–13 mm in diameter, or ellipsoid, 10–12 × 7–8 mm	2–8 in clusters, densely covered with tiny protuberances, globose, 9–11 mm in diam.

### Phylogenetic analysis

**Figure 1.**
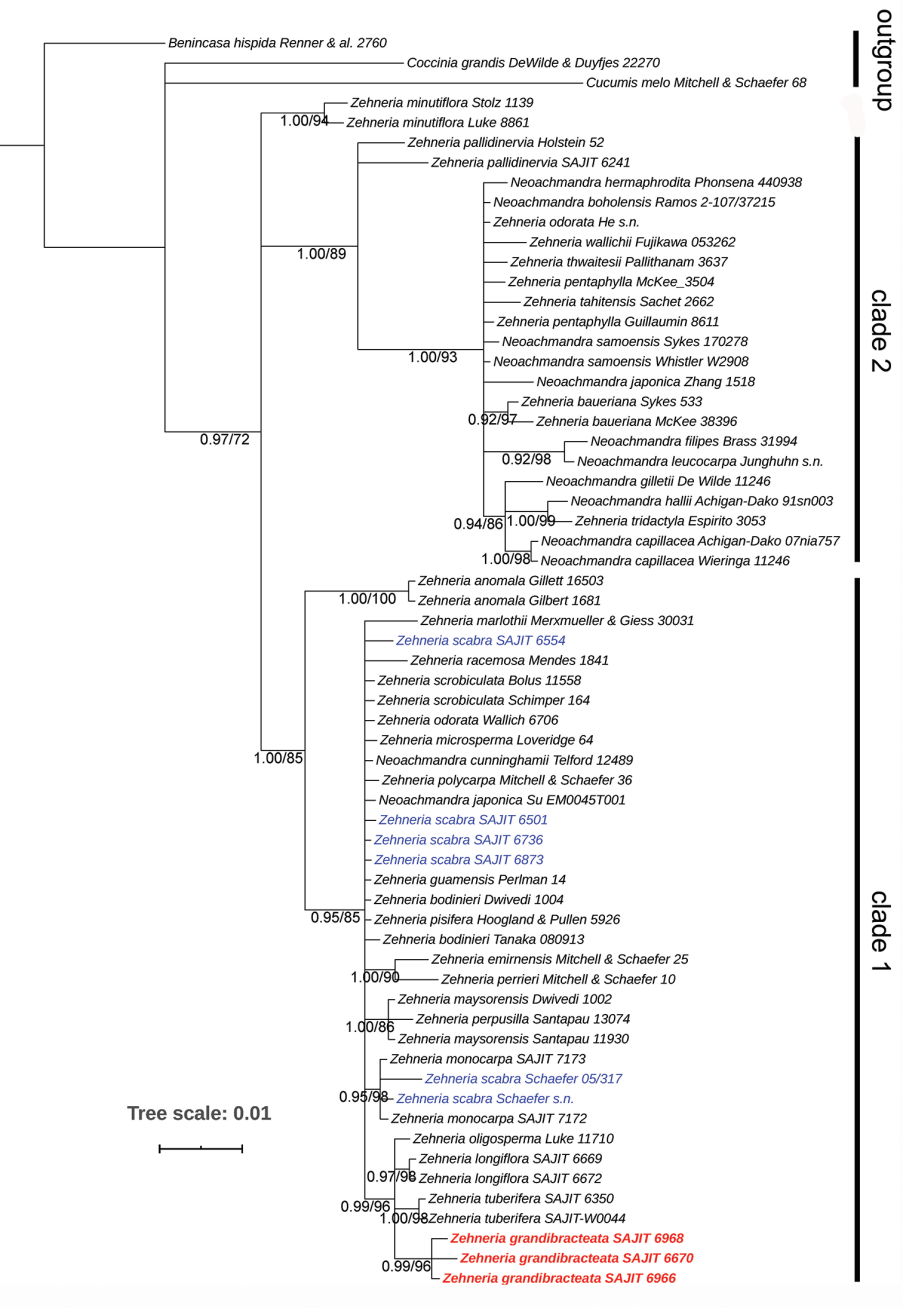
Bayesian tree inferred from the nrITS sequences dataset to elucidate the phylogenetic position of *Zehneria grandibracteata*. Bayesian posterior probability values > 0.9 and bootstrap values ≥ 70% are shown below the branches. The new species is highlighted in bold and red colour and *Z. scabra* is noted in blue colour.

In total, 60 sequences representing 38 *Zehneria* species were included in our dataset. Multiple sequences per species were identical as to some species, like *Z. grandibracteata*, *Z. anomala*, *Z. tuberifera* and *Z. longiflora*. They might, however, be different regarding the other species, such as *Z. scabra*, Z. *pallidinervia* and *Z. minutiflora*. The final trimmed alignment of 63 sequences has 721 columns, with 92 parsimony-informative sites. *Z. grandibracteata* differs in the 71^th^ position (G vs. A) and 208^th^ position (A vs. T) of ITS1 alignment from other *Zehneria* species. HKY+F+G4 was selected as the best-fit model to infer the Maximum Likelihood tree and Bayesian tree. As shown in Figure 1, three accessions of *Z. grandibracteata* clustered together with robust support (PP = 0.99; BS = 98%). Then, it joined the other three East African taxa group (*Z. oligosperma*, *Z. tuberifera* and *Z. longiflora*), which offers morphological synapomorphies and a conclusive biogeographic scenario of its evolution. These four species formed a monophyly with high support (PP = 0.99; BS = 96%). However, accessions of *Z. scabra* did not form a monophyly as expected (newly-sequenced accessions are monophyletic, but two previously-published accessions are nested in *Z. monocarpa*). Despite the new species being closely related to *Z. scabra*, they are not recognised as monophyletic in our phylogenetic tree.

### Taxonomic description

#### Zehneria grandibracteata

Taxon classificationPlantaeCucurbitalesCucurbitaceae

G.W. Hu, Neng Wei & Q.F. Wang sp. nov.

E886B507-9207-58FD-800E-021876EA4B7A

urn:lsid:ipni.org:names:77212572-1

Figures 3 , 4

##### Diagnosis.

It is close to *Z. scabra*, but differs by its consistently ovate leafy probracts (linear minute or even absent in *Z. scabra*), subglabrous condition of the entire plant (puberulous in *Z. scabra*), shorter receptacle-tube (1.8–3 mm long vs. 2–5.5 mm in *Z. scabra*) and filaments (ca. 1.5 mm long vs. 1–2.5 mm in *Z. scabra*), as well as sessile and denser inflorescences (cluster of 8–30 in male, 6–22 in female vs. 2–60 in male, 1–16 in female in *Z. scabra*) (Table 2).

##### Type.

Kenya. Nandi County, South Nandi Forest, Morongiot area, 0°04'N, 35°00'E, elev. 1980 m, 20 April 2018, *Sino-Africa Joint Investigation Team* (*SAJIT*) *006973* (Female) (holotype HIB!; isotype EA!, HIB!)

##### Description.

Perennial climber, 8 m or longer; rhizome robust, woody when old, up to 2.5 cm in diam., roots slender, branched; stem many-branched, grooved, usually contorted when aged, sparsely puberulous except densely hairy at nodes. Leaves simple, petioles 2–7 cm long, grooved adaxially, subglabrous; blades 38–65 × 28–46 mm, ovate-cordate in outline, shallowly 3-lobed occasionally, membraneous, subglabrous, deeply cordate to subtruncate at base, margin slightly sinuate-toothed, apex acuminate and apiculate; scabrid-punctate above, 3–11 main veins sunken adaxially and protrudent abaxially, with sparsely-scattered bristles on both sides, especially on veins and margins; tendrils simple, up to 15 cm long. Dioecious. Inflorescence base with a well-developed leafy probract, up to 18 × 12 mm, ovate, incurved, beak-like, persistent, 2–3 main veins from base, base cordate, apex acuminate. Male inflorescences axillary, sessile, subumbelliform, 8- to 30-flowered, pedicels 3–12 mm long; receptacle-tube 1.8–3 mm long, campanulate, greenish-cream, turning into orange when aged, inner surface densely hairy, outside surface glabrous; sepal lobes 5, ca. 1 mm long, dentiform, pale green; petal lobes 5, ca. 1.8 × 1.5 mm, triangular-ovate, white, turning cream to orange when aged. Stamens 3, inserted in middle of tube; filaments ca. 1.5 mm long, subglabrous, lower half fused with tube; anthers ca.1 mm long, ellipsoid, 2-thecae; thecae 1 mm long, vertical, slightly curved, connective elliptic, with finely papillose hairs; disc ca. 1 mm in diam., depressed globose, obscurely trilobed, elevated. Female inflorescences axillary, sessile, 6- to 22-flowered in umbelliform clusters; pedicel 4–6 mm long; perianth similar to male flowers; ovary subglobose, glabrous, with evident neck up to 1 mm long; style 2–3.5 mm long, glabrous, stigma ca. 1.5 mm in diam., with 3 down-curved papillose lobes; staminodes 3, ca. 1.5 mm long, linear, glabrous, at base of the tube; disc ca. 1.8 mm in diam., annular, 3-lobed, surrounding base of style, free from tube. Fruits clustered, 8–10 mm in diam., subglobose, subglabrous, sparsely covered with tiny protuberances, turning from green to orange when mature; pedicel 5–10 mm long. Seed ovate in outline, narrowly bordered, lenticular, compressed.

##### Distribution and ecology.

Numerous populations of this new species have been documented in the western parts of Kenya’s forests, including Morongiot and Kobujoi areas of South Nandi Forest, Kapsasur area of Nandi Centre, Yale River Trail of Kakamega Forest, Timbilil and Sambret Catchment area of south-western Mau Forest. It usually climbs over tree trunks or twines around shrubs in moist forests or at forest margin at elevations of 1950–2230 m.

##### Conservation status.

This new species was found in the western Kenyan forests with numerous localities. It is locally quite common in the wild and frequently grows in forests or at forest margins. Thus, we assess it to be “Least Concern” (LC) based on IUCN Red List Categories and Criteria (IUCN 2001).

##### Phenology.

Flowering and fruiting from April to July and November to January, corresponding to the wet seasons of the bimodal rainfall pattern of this region.

##### Etymology.

The epithet “*grandibracteata*” refers to the fairly large leafy probract of this new species.

##### Additional specimens examined

**(Paratypes).** Kenya. Nandi County, South Nandi Forest, Kobujoi area, 34°57'E, 0°04'N, elev. 1970 m, 11 December 2016, *SAJIT 006670* (EA! HIB!); Nandi County, South Nandi Forest, Morongiot area, 0°04'N, 34°55'E, elev. 1980 m, 19 April 2018, *SAJIT 006966* (EA! HIB!) and *SAJIT 006968* (EA! HIB!); Nandi County, Nandi Centre, Kapsasur area, elev. 1970 m, 18 April 2018, *SAJIT s.n.* (HIB!); Kakamega County, Kakamega Forest, Yale River Trail, 0°16'N, 34°52'E, 7 January 2017, *SAJIT s.n.* (HIB!); Kericho County, Changana Tea Estate, 5.3 miles south of Kericho Town, 0°27'S, 35°18'E, 22 November 1967, *Perdue R.E. and Kibuwa S.P. 9179* (BR! EA! K!); Kericho County, Sambret Catchment of southwestern Mau Forest, 0°22'S, 35°23'E, 2160 m, 5 July 1962, *Kerfoot O. 3375* (EA! K!); Kericho County, Sambret Catchment of Southwestern Mau Forest, 0°26'S, 35°22'E, 2230 m, 16 Jan 1963, *Kerfoot O. 4696* (EA!); Kericho County, Timbilil of southwestern Mau Forest, 0°18'S, 35°31'E, 2130 m, Jan 1963, *Kerfoot O. 4708* (EA!).

## Discussion

**Figure 2.**
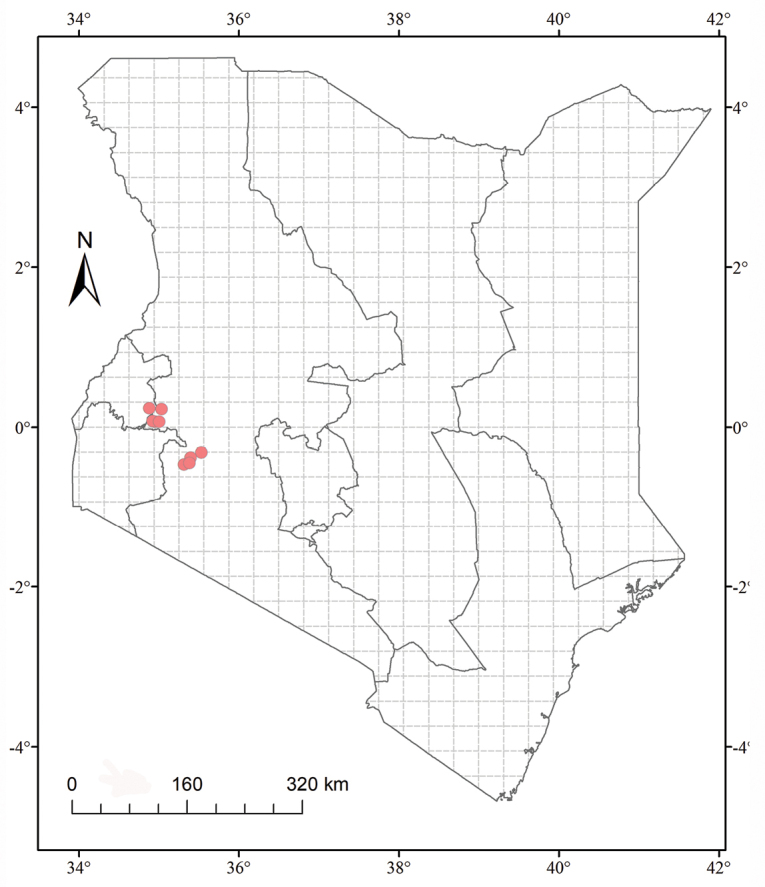
Distribution map of *Zehneria grandibracteata* in Kenya. Red dots indicate its documented localities.

Our *Z. grandibracteata* collections are recognised as monophyletic, separated from the related *Z. scabra*. The possible reasons to explain the paraphyly of *Z. scabra* in our phylogeny are 1) the nrITS provides limited phylogenetically-informative sites in *Zehneria* and mutations on few loci produced inconsistent phylogenetic topology; 2) the two accessions collected by Schaefer here probably should be *Z. monocarpa*, which was separated from *Z. scabra* recently (Ngumbau et al. 2020). Furthermore, we also found that species of *Neoachmandra* in the sense of De Wilde and Duyfjes (2006a) and De Boer et al. (2015), are paraphyly. In line with the conclusion made by Dwivedi et al. (2018), the whole genus tended to be separated into two major clades (clade 1 and clade 2), with African taxa being the basal lineages. Even though the morphological characters proposed by De Wilde and Duyfjes (2006a) are not suitable for splitting groups (Dwivedi et al. 2018), they are still important and helpful characters when identifying at the species level. The ovate leafy probracts in our new species are readily distinguishable, while probracts on other East African taxa tend to be minute linear hooked or even caducous. Geographically, it is only documented in western Kenyan forests (Figure 2), while *Z. scabra* is widely distributed in the pantropical Old World region. Furthermore, the molecular phylogenetic analysis of *Zehneria*, based on nrITS sequences, also supports the segregation of *Z. grandibracteata* from *Z. scabra*. Combined with morphological and phylogenetic analyses, *Z. grandibracteata* is confirmed as new to science.

**Figure 3.**
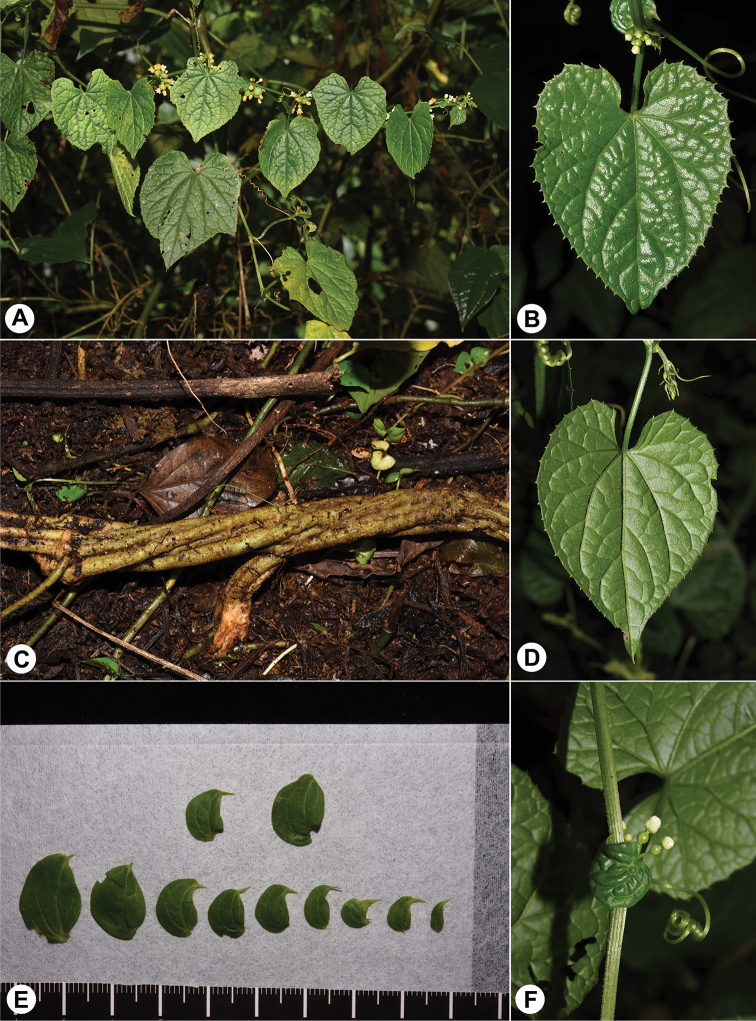
Photographs showing vegetative characters of *Zehneria grandibracteata***A** climbing stem of female plant in habitat **B** adaxial lamina **C** creeping stem **D** abaxial lamina **E** probracts at different developing stages **F** tendril and probract at base of female inflorescence. Scale in picture **E** represents cm.

The broadly circumscribed concept of *Zehneria* may represent a better natural group, while there is no comprehensive classification system for this group until now. Jeffrey (1962) tried to divide Zehneria into two subgenera, namely subg. Zehneria and subg. Pseudokedrostis (Harms 1923: 616) Jeffrey (1962: 368) (largely accord with clade 1 and clade 2 here), mainly based on the position of stamen insertion, the thecae and connective of anther and length of pedicel. Viewing from the phylogenetic tree inferred by Dwivedi et al. (2018), as well our tree here, Jeffrey’s morphological summaries mostly work well. Besides, the two fruit shapes, short (sub)globose and long fusiform/ellipsoid, largely fit in with clade 1 and clade 2, respectively, though several taxa with round fruits could also be found in clade 2. All these characters would provide insights into building a classification system within the genus *Zehneria*. Future biogeographical analysis, based on a robust phylogenic framework, would substantially improve our understanding towards its origin and dispersal history.

**Figure 4.**
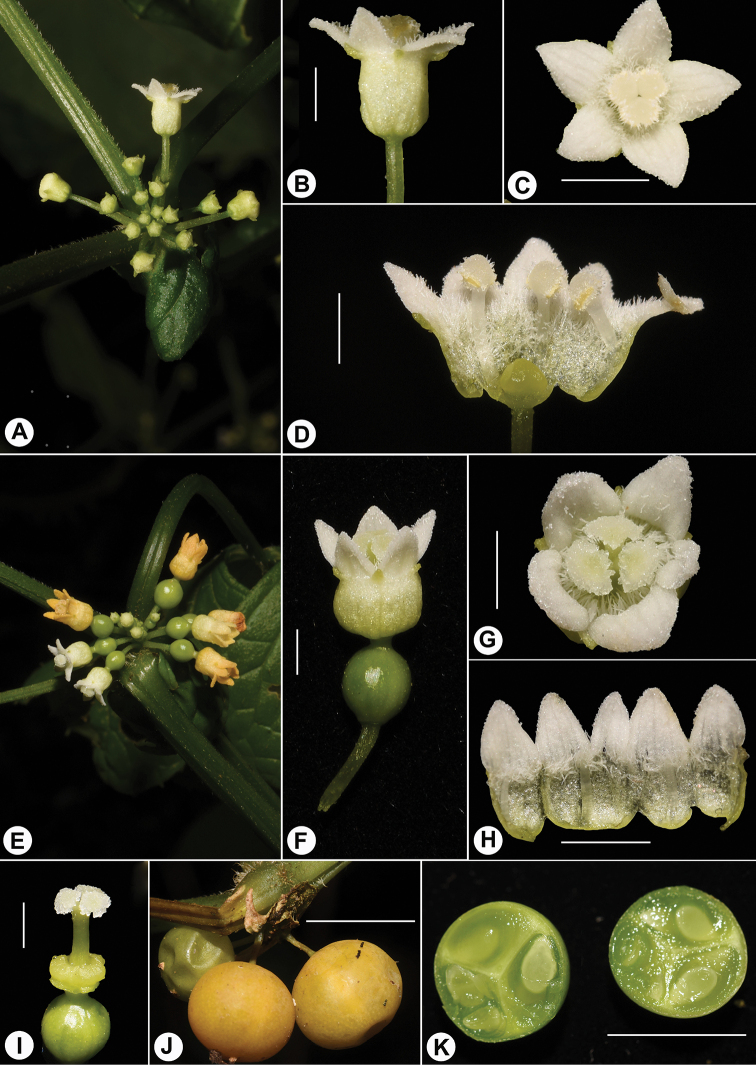
Photographs showing reproductive characters of *Zehneria grandibracteata***A** male inflorescence **B** male flower, side view **C** male flower, top view **D** dissected male flower showing disc and stamens **E** female inflorescence **F** female flower, side view **G** female flower, top view **H** dissected female flower showing staminodes **I** pistil and disc **J** infructescence **K** cross-section of fruit. Scale bars: 2 mm (**B–D, F–I**); 1 cm (**J, K**).

## Supplementary Material

XML Treatment for Zehneria grandibracteata

## Supplementary materials

Supplementary material 1 Modified CTAB protocol on the base of Doyle and Doyle (1987) Data type molecular data https://binary.pensoft.net/file/468716This dataset is made available under the Open Database License (http://opendatacommons.org/licenses/odbl/1.0/). The Open Database License (ODbL) is a license agreement intended to allow users to freely share, modify, and use this Dataset while maintaining this same freedom for others, provided that the original source and author(s) are credited.Neng Wei, Zhi-Xiang Zhong, David Kimutai Melly, Solomon Kipkoech, Benjamin Muema Watuma, Veronicah Mutele Ngumbau, Peris Kamau, Guang-Wan Hu, Qing-Feng Wang
