# Supplementary material for: Zehneria grandibracteata (Cucurbitaceae), an overlooked new species from western Kenyan forests
Source: PhytoKeys. 2020 Oct 28;165:85–98. doi: 10.3897/phytokeys.165.57399 (PMC7642130; doi:10.3897/phytokeys.165.57399)
Supplement: Supplementary material 1 — Modified CTAB protocol on the base of Doyle and Doyle (1987) [file phytokeys-165-085-s001.docx]

**Modified CTAB protocol on the base of Doyle and Doyle (1987)**

Prior to normal extraction procedures of CTAB protocol, a impurity washing buffer solution for removing the interferent was used to wash the mashed powder after grinding. The formula is below.

Impurity washing buffer solution:

100 mmol/L Tris-HCl, 5 mmol/L EDTA, 5% Glycerinum, 10% PEG8000, 0. 1% Tris ( 2-carboxyethyl) phosphine hydrochloride (TCEP)

**Materials**

CTAB buffer

Microfuge tubes

Mortar and Pestle

Liquid Nitrogen

Microfuge

Absolute Ethanol (ice cold)

70 % Ethanol (ice cold)

7.5 M Ammonium Acetate

55℃ water bath

Chloroform : Iso Amyl Alcohol (24:1)

ddH_2_O

Agarose

6x Loading Buffer

1x TBE solution

Agarose gel electrophoresis system

Ethidium Bromide solution

CTAB buffer 100ml:

2.0 g CTAB (Hexadecyl trimethyl-ammonium bromide)

10.0 ml 1 M Tris pH 8.0

4.0 ml 0.5 M EDTA pH 8.0 (EthylenediaminetetraAcetic acid Di-sodium salt)

28.0 ml 5 M NaCl

40.0 ml H_2_O

1 g PVP 40 (polyvinyl pyrrolidone (vinylpyrrolidine homopolymer) Mw 40,000)

Adjust all to pH 5.0 with HCl and make up to 100 ml with H_2_O.

1 M Tris pH 8.0:

Dissolve 121.1 g of Tris base in 800 ml of H_2_O. Adjust pH to 8.0 by adding 42 ml of

concentrated HCL. Allow the solution to cool to room temperature before making the

final adjustments to the pH. Adjust the volume to 1 L with H_2_O. Sterilize using an

autoclave.

5x TBE buffer:

54 g Tris base

27.5 g boric acid

20 ml of 0.5M EDTA (pH 8.0)

Make up to 1L with ddH_2_O.

To make a 0.5x working solution, do a 1:10 dilution of the concentrated stock.

**Procedure**

- Grind 200 mg of plant tissue and add 1ml impurity washing buffer solution, mix thoroughly. Spin the mixture at 12000 g for 1 min, discard the supernatant.

- Add 500 μl of CTAB buffer and mix thoroughly.

- Incubate the CTAB/plant extract mixture for about 30 min at 55℃ in a recirculating

water bath.

- After incubation, spin the CTAB/plant extract mixture at 12000 g for 5 min to spin

down cell debris. Transfer the supernatant to clean microfuge tubes.

- To each tube add 250 μl of Chloroform : Iso Amyl Alcohol (24:1) and mix the

solution by inversion. After mixing, spin the tubes at 13000 rpm for 1 min.

- Transfer the upper aqueous phase only (contains the DNA) to a clean microfuge

tube.

- To each tube add 50 μl of 7.5 M Ammonium Acetate followed by 500 μl of ice cold

absolute ethanol.

- Invert the tubes slowly several times to precipitate the DNA. Generally the DNA can

be seen to precipitate out of solution. Alternatively the tubes can be placed for 1 hr at

-20℃ after the addition of ethanol to precipitate the DNA.

- Following precipitation, the DNA can be pipetted off by slowly rotating/spinning a

tip in the cold solution. To wash the DNA, transfer the precipitate into a microfuge

tube containing 500 μl of ice cold 70 % ethanol and slowly invert the tube. Repeat.

- After washing, spin the DNA into a pellet by centrifuging at 13000 rpm for 1 min.

Remove all the supernatant and allow the DNA pellet to dry (approximately 15 min).

- Resuspend the DNA in 100μl ddH_2_O. RNaseA (10 μg/ml) can be added to the water prior to dissolving the DNA to remove any RNA in the preparation (10 μl RNaseA in 10ml H_2_O).

- After resuspension, the DNA is incubated at 65℃ for 20 min to destroy any DNases

that may be present and store at 4℃.

- Spectrophotometry will give an indication of the concentration and cleanliness for DNA extractions.
